# Structural Basis of Eco1-Mediated Cohesin Acetylation

**DOI:** 10.1038/srep44313

**Published:** 2017-03-14

**Authors:** William C. H. Chao, Benjamin O. Wade, Céline Bouchoux, Andrew W. Jones, Andrew G. Purkiss, Stefania Federico, Nicola O’Reilly, Ambrosius P. Snijders, Frank Uhlmann, Martin R. Singleton

**Affiliations:** 1 Structural Biology of Chromosome Segregation Laboratory, The Francis Crick Institute, 1 Midland Road, London NW1 1AT, UK; 2 Chromosome Segregation Laboratory, The Francis Crick Institute, 1 Midland Road, London NW1 1AT, UK; 3 Protein Analysis and Proteomics Platform, The Francis Crick Institute, 1 Midland Road, London NW1 1AT, UK; 4 Structural Biology Platform, The Francis Crick Institute, 1 Midland Road, London NW1 1AT, UK; 5 Peptide Chemistry Laboratory, The Francis Crick Institute, 1 Midland Road, London NW1 1AT, UK

## Abstract

Sister-chromatid cohesion is established by Eco1-mediated acetylation on two conserved tandem lysines in the cohesin Smc3 subunit. However, the molecular basis of Eco1 substrate recognition and acetylation in cohesion is not fully understood. Here, we discover and rationalize the substrate specificity of Eco1 using mass spectrometry coupled with *in-vitro* acetylation assays and crystallography. Our structures of the *X. laevis* Eco2 (xEco2) bound to its primary and secondary Smc3 substrates demonstrate the plasticity of the substrate-binding site, which confers substrate specificity by concerted conformational changes of the central β hairpin and the C-terminal extension.

The topological entrapment of chromosomes by cohesin (Smc1, Smc3, Scc1, and Scc3) is central to genome integrity^1^,^2^,^3^. Cohesin is loaded onto chromatin by the Scc2-Scc4 loader complex^4^,^5^,^6^, however persistent cohesion cannot be established without the acetylation of two conserved tandem lysines on the Smc3 subunit by the Eco1 acetyltransferase (ACT)^7^,^8^,^9^,^10^. Cohesin release from DNA relies on the interaction between DNA and these conserved lysines^11^. Acetylation of these lysines antagonizes Wapl-mediated cohesin release by blocking DNA-induced ATP hydrolysis and the dissociation of the Smc3/Scc1 interface, thus locking cohesin onto chromatin^11^,^12^,^13^,^14^,^15^.

Eco1 is an acetyltransferase that belongs to the Gcn5-related N-acetyltransferase (GNAT) family^16^,^17^. Unlike other GNAT family members, Eco1 contains a zinc-finger (ZnF) domain that is similar to those found in the Moz/Ybfs/Sas2/Tip60 (MYST) family of histone acetyltransferases (HATs). The ZnF is responsible for HATs recognizing nucleosome in histone tail acetylation^18^,^19^,^20^, while in Eco1, the ZnF enhances its acetyltransferase activity during sister chromatid cohesion^21^. Its S-phase localization to the replication fork is thought to be via a direct interaction with PCNA^22^,^23^,^24^.

As well as its function in establishing replication-coupled cohesion, Eco1 has also been proposed to act during double-strand break repair. Break-induced phosphorylation of Scc1 is thought to trigger its Eco1-mediated acetylation, which also antagonizes Wapl releasing activity^25^. Furthermore, Eco1 is regulated through Cdk1-mediated phosphorylation, which promotes its SCF^Cdc4^-dependent ubiquitination and degradation after S phase^26^. Interestingly, the recruitment of xEco2, the primary cohesin ACT in *Xenopus laevis,* onto chromatin was dependent on the pre-RC assembly but independent of cohesin loading and DNA synthesis^27^.

Like cohesin and its regulatory subunits, Eco1 homologues also have important implications in human developmental disorders^28^,^29^. Human Esco1 (hEsco1) is enriched at sites occupied by cohesin and CTCF, whereas human Esco2 is targeted to genes controlled by RE-1 silencing transcription factor (REST), implying the cohesin ACTs’ roles in gene regulation^30^. In fact, the loss of human Esco2 results in SC Phocomelia and Roberts Syndrome (RBS)^31^,^32^, while in yeast a RBS mutant equivalent of Eco1 reduces ribosomal DNA (rDNA) transcription^33^.

Despite the importance of acetylation in cohesion establishment and human diseases, little is known about how Eco1 targets its canonical substrate Smc3 and how this acetylation stabilizes cohesin on DNA. To provide further insights into substrate recognition by Eco1, we reconstitute an *in-vitro* acetylation assay and coupled it with mass spectrometry (MS) analyses to determine both the dynamics and substrate specificity of Eco1-mediated acetylation. In our assays, we observe a fast acetylation event of K112 occurring prior to the acetylation of K113. By aligning the sequences of different acetylated cohesin peptides, we show that the target lysine of acetylation is favoured by the flanking of an aliphatic residue and an acidic/polar residue, and that this motif can be recognized in a bidirectional fashion. Furthermore, we present the crystal structures of *Xenopus laevis* Eco2 (xEco2) ACT domain bound to two different substrate peptides, which reveal that Eco1 substrate specificity is determined by the concerted conformational changes of the conserved central β hairpin and the C-terminal extension (C extension).

## Results and Discussion

### *S. cerevisiae* Eco1 Acetylates Smc3 K112 Prior to K113

**Figure 1:**
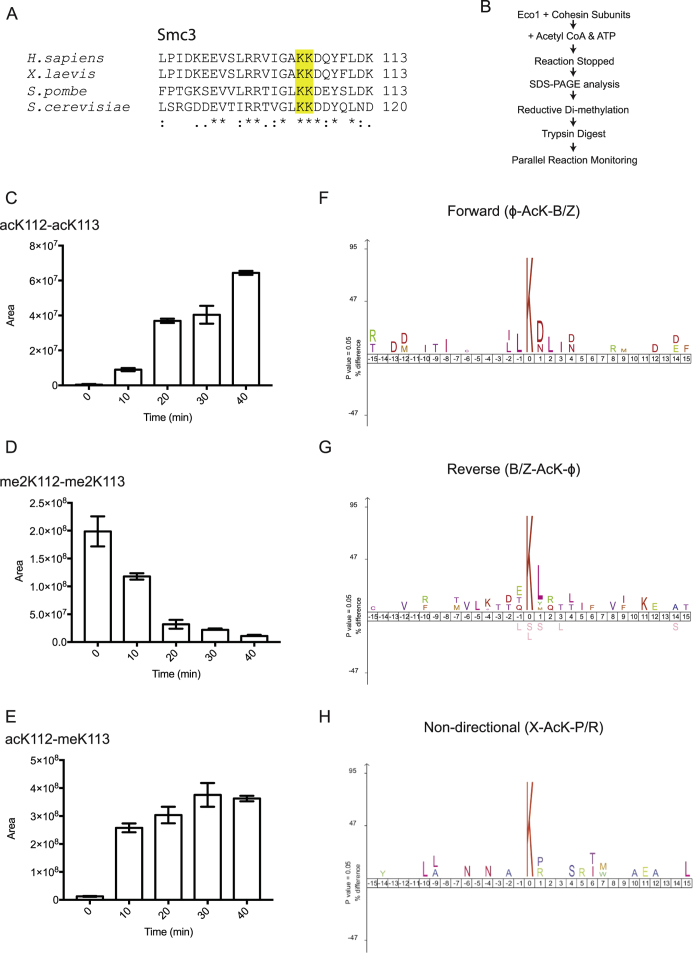
Smc3 K113 is the primary target for Eco1 and Eco1 substrate motif consensuses. (**A**) Sequence alignment of Smc3 β hairpin region with the conserved tandem lysines highlighted. (**B**) Schematic of *in-vitro* Eco1-mediated acetylation time-course assay coupled to MS analysis. (**C**) *In-vitro* Eco1-mediated acetylation assay showing increased level of tandem lysine (K112 & K113) acetylation peptide over time. Area is in arbitrary unit. (**D**) *In-vitro* Eco1-mediated acetylation assay showing decreased level of unacetylated (dimethylated on both K112 and K113) peptide over time. (**E**) *In-vitro* Eco1-mediated acetylation assay showing rapid increase of sole- K113 acetylation peptide from 0 min to 10 min and approaching completion over time. This suggests that a faster targeting of K112 is followed by a slower acetylation event of K113. (**F**) Eco1 substrate motif consensus in forward direction (n = 36) indicating target-lysine flanked by an aliphatic residue at P − 1 and an acidic/polar residue at P + 1. (**G**) Eco1 substrate motif consensus in reverse direction (n = 42) indicating target-lysine flanked by an acidic/polar residue at P − 1 and an aliphatic/hydrophobic residue at P + 1. (**H**) Non-directional Eco1 substrate motif consensus (n = 27) indicating target-lysine flanked by proline residues (P − 1 and P + 1) or a basic residue at P + 1.

Previous studies in yeast have shown that Eco1-mediated acetylation of the conserved tandem lysine motif (*S. cerevisiae* K112/K113; human K105/106) in Smc3 (Fig. 1A) is important in establishing sister-chromatid cohesion during DNA replication^7^,^9^. In human Smc3, a non-acetylatable human K106R mutation, but not K105R, of the tandem lysine motif was the primary cause of sister chromatid cohesion loss^8^. To study the relative importance of the two conserved lysines, we reconstituted an *in-vitro* Eco1-mediated acetylation time-course assay using recombinantly purified *S. cerevisiae* cohesin and Eco1 (Fig. 1B).

Reaction products were separated by SDS-PAGE, with Smc3-containing gel bands subsequently subjected to an in-gel reductive di-methylation step^34^. As a result of the enzymatic and chemical reactions, K112 and K113 were either acetylated or di-methylated at all time points. This strategy facilitated our analysis since K112 and K113 are blocked for trypsin proteolysis and hence the peptide TVGLKKDDYQLNDR could be consistently extracted from the gel prior to mass spectrometry analysis. A Parallel Reaction Monitoring (PRM) mass spectrometry experiment was used to target three different peptide masses corresponding to the four different variants of the target peptide. To distinguish the isobaric variants acK112-meK113 and meK112-acK113 the intensities of the y9 and b5 ion were monitored in the Higher Energy Collisional Dissociation (HCD) fragmentation spectra (Supplementary Fig. S1B and S1C)^35^. In the same run, we also measured the peptide signal prior to fragmentation for the three different intact peptide masses using a 4 Dalton window Selected Ion Monitoring (SIM) scan. Inspection of the PRM data revealed the presence of high y9 and b5 ion intensities for the acK112-meK113 variant at time points 10–40 but not at time point 0 points (Supplementary Fig. S1B). The y9 and b5 ion intensities for the meK112-acK113 variant were absent at all time-points and hence we used the SIM peak areas to quantify the acK112-meK113 variant throughout the time course.

The level of acK112-meK113 increased rapidly between 0 min to 10 min then approached saturation (Fig. 1E). Our data also showed that there was a general increase in the abundance of the peptide containing both K112 and K113 acetylation (acK112-acK113) over time (Fig. 1C). This confirms that our purified Eco1 can di-acetylate cohesin *in vitro*. As expected, a reverse trend was observed for the non-acetylated peptide variant (Fig. 1D). Our results reveal that K112 acetylation happens prior to the slower K113 acetylation, which appears to be more important for cohesin establishment, at least in mammalian systems^8^.

Interestingly, such tandem (acetyl)-lysine motifs are also substrates for deacetylation by the Sirtuins, Sirt1-3 where a similar position-dependent rate dependence is observed and the acetylation state of one lysine may affect the rate of the adjacent deacetylation^36^. However, it is not clear if these proximity mechanisms can be generalized to acetyltransferases as well.

### Eco1 Substrate Motif Consensuses

Apart from Smc3 acetylation, Eco1 also targets itself for auto-acetylation^8^,^25^,^37^. DNA double-strand break (DSB)-induced cohesion in yeast is also thought to require the acetylation of K84 and K210 in the cohesin subunit Scc1^25^ and a recent study has shown that DNA lesions can induce Eco1-mediated acetylation of PCNA^38^. The auto-acetylation activity and the lack of any known substrate motif amongst cohesin targets suggest that Eco1 has relatively broad substrate specificity. To try and identify the molecular determinants of preferred substrates, we performed *in-vitro* Eco1-mediated acetylation with recombinantly purified cohesin core complex and regulatory subunits (Smc1-Smc3-Scc1-Scc3, Scc2-Scc4, Pds5, and Wapl, all from *S. cerevisiae*) and subjected the reaction products to MS analysis (Fig. 1B). After subtracting background acetylation that was not due to Eco1 (see Materials and Methods), we discovered that the acetylated lysine had a propensity to be flanked by an aliphatic (ϕ) residue and an acidic/polar (B/Z) residue in all cohesin subunits (Supplementary Table S1). However, Eco1 can acetylate lysines with flanking residues in either direction, suggesting Eco1 substrate recognition is bidirectional. Based on this finding, we generated three Eco1 substrate motif consensuses using the iceLogo server^39^ by aligning 36 peptides in the forward direction (ϕ-AcK-B/Z) (Fig. 1F), 42 peptides in the reverse direction (B/Z-AcK-ϕ) (Fig. 1G), and 27 peptides without any clear directionality (X-AcK-P/R) from our MS analysis (Fig. 1H). Peptides that are categorized as non-directional favor a proline (P) or an arginine (R) as the flanking residue, thus conforming to the β hairpin conformation of the Smc3 tandem lysines in the Smc3-Scc1N structure^40^. Interestingly, it has been proposed that Chk1-dependent phosphorylation of Scc1 on S83 stimulates Eco1-mediated acetylation on K84^25^. This phosphoserine at P − 1 of K84 could mimic the flanking acidic residue that we identify in our “reverse” substrate alignments (Fig. 1F and G). The target motif of another identified Eco1 substrate, Mps3 also conforms to this reverse pattern, where two of the three acetylated lysines have a leucine or tryptophan in the P + 1 position, and the third a glutamate in the P − 2 position^41^, while the recently identified Eco1-targetted target lysine K20 in PCNA fits the “forward” consensus with a phenylalanine in the P − 1 and aspartate at P + 1 positions^38^. We also note that in Smc3, acetylation of the K112 amino group ensures that K113 is then in a neutral (P − 1), acidic (P + 1) environment which is more resembles the “forward” consensus than the unmodified K112, which would put K113 in a basic (P − 1), acidic (P + 1) context, which appears highly disfavored in our analysis.

Our results suggest that the sequence context of the target lysine is important for substrate discrimination, and the consensus motifs we identify may be of use in searching for novel Eco1 targets. Under cellular conditions, where the enzyme concentration is much lower than that employed in our *in vitro* analysis, other targeting and regulatory mechanisms doubtless assist substrate selection such as temporal and spatial localization of the enzyme^23^,^24^,^26^,^30^,^42^,^43^.

### Structures of xEco2 and xEco2-Substrate Complexes

**Figure 2:**
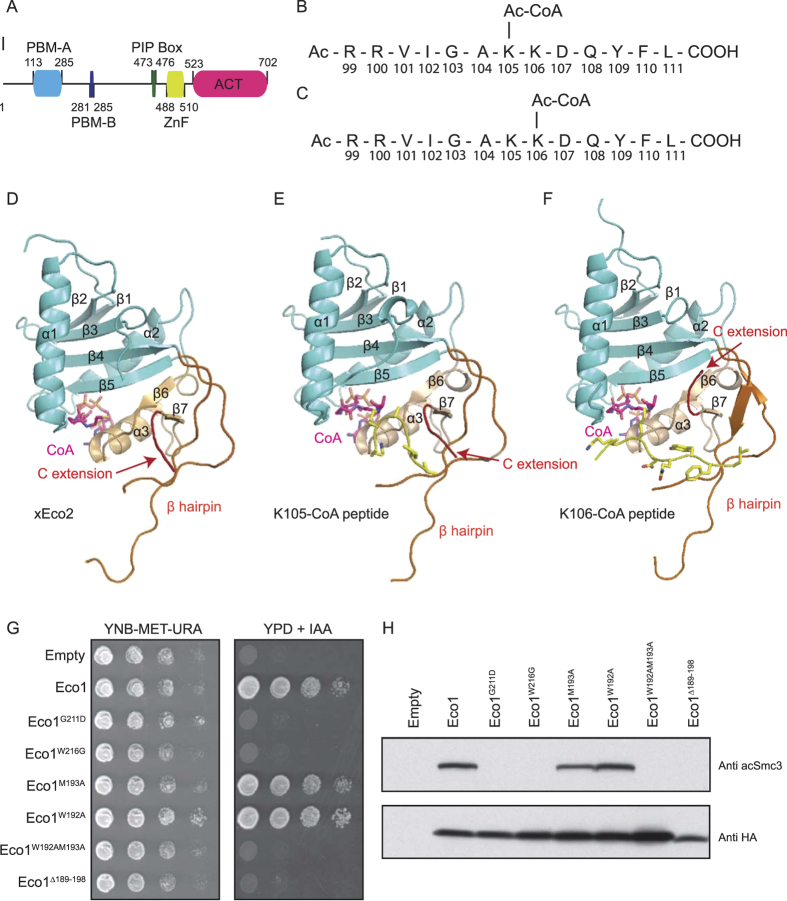
Structures of the xEco2-K106-CoA and xEco2-K105-CoA complexes. (**A**) Schematic of xEco2 indicating the zinc-finger (ZnF) domain and the construct boundary of the acetyltransferase (ACT) domain. PIP box – PCNA interaction peptide, PBM-A/B as defined in Higashi *et al*.^27^. (**B**) Design of the Eco1 substrate with a 13-residue Smc3 peptide conjugated with CoA at K105 (K105-CoA). (**C**) Design of the Eco1 substrate with a 13-residue Smc3 peptide conjugated with CoA at K106 (K106-CoA). (**D**) Structure of the xEco2 ACT. The xEco2 ACT consists of an N-terminal domain (cyan) with a central β hairpin (orange) and a C-terminal domain (wheat) with a conserved C extension (red). (**E**) Structure of the xEco2 ACT bound to K105-CoA (yellow). The K105-CoA peptide adopts a β turn conformation and is coordinated by the C extension as a contiguous β sheet. The xEco2 ACT has the same colouring scheme as in (**D**). (**F**) Structure of the xEco2 ACT bound to K106-CoA (yellow). The K106-CoA peptide is coordinated in a β strand conformation by the β hairpin. The xEco2 ACT has the same colouring scheme as in (**D**) with the C extension (red) positioning upwards to allow the full accommodation of the K106-CoA peptide. (**G**) Yeast survival assays comparing wild-type and mutant Eco1 strains. *ECO1* mutants were tested for the ability to restore viability to a strain carrying an *ECO1* degron allele. Cells of each strain were serially diluted and spotted on synthetic minimal medium without methionine (CSM – Met) plates or on plates containing indoleacetic acid (IAA). (**H**) *In-vivo* acetylation assays using anti-acetyl Smc3 antibody. Expression levels of Eco1 mutants were monitored by anti-HA antibody. Western blots have been cropped for presentation.

The variation in acetylation rates seen in our MS data (Fig. 1C and E, and Supplementary Fig. S1A and S1B) is likely due to the different modes of substrate recognition by Eco1 towards the tandem lysines (*S. cerevisiae* K112/K113; *X. laevis* K105/K106). To gain insights into Smc3 recognition by Eco1, we performed crystallographic studies on the *Xenopus laevis* Eco2 (xEco2) ACT domain (residues 523–702) (Fig. 2A and Supplementary Fig. S2A), with and without the binding to its canonical Smc3 substrate peptides. The ACT domains of xEco1 and xEco2 show a high degree of homology with 60% sequence identity in this region and are generally highly conserved throughout evolution^22^. The biological differences between the orthologs are thought to result from the non-conserved N-terminus, not included in our structure^27^,^44^. The affinity between Eco1 and cohesin is low as pull-down assays with recombinantly purified yeast proteins were unable to show any detectable interaction (data not shown). To circumvent this problem, we exploited the strong binding of CoA to ACTs and synthesized two 13-residue Smc3 (residues 99–111) peptide-CoA conjugates (K105-CoA and K106-CoA) each containing an isopropionyl bridge^45^,^46^: peptide K105-CoA is conjugated with CoA at K105 whereas peptide K106-CoA has CoA conjugated to K106 (Fig. 2B and C). Crystals of the peptide-free xEco2 (henceforth xEco2) diffracted to 3.0 Å, whereas the binding of the K105-CoA and K106-CoA peptides to xEco2 improved the resolution to 2.4 and 1.9 Å respectively (Supplementary Table S2).

The structures of xEco2, xEco2-K105-CoA, and xEco2-K106-CoA were determined by molecular replacement using the crystal structure of human Esco1 ACT (hEsco1) (PDB: 4MXE)^47^ as a search model (Supplementary Table S2) (Fig. 2D,E,F, and Supplementary Fig. S3). The xEco2 ACT contains a mixed α/β structure with a globular fold structurally resembling the homology fold of those in the GNAT family. The xEco2 ACT fold is divided into two domains: the N-terminal domain contains α helix 1 and 2, and β strand 1–5 with a conserved central β hairpin insertion between β4 and β5 that is unique to cohesin ACTs; the C-terminal domain contains α helix 3, and β strand 6 and 7. The binding site for CoA is sandwiched between the N- and C-terminal domains involving β5 and α3, while the substrate peptides binds mainly to the central β hairpin and the C-terminal domain including the conserved C-terminal extension (C extension). On β5 of the N-terminal domain, we locate G635 as an Eco1 yeast-equivalent temperature-sensitive mutation G211D and a Robert Syndrome mutation W640G (*S. cerevisiae* W216G; human W539G) (Supplementary Fig. S2A and Fig. S2B). Mutations of these buried residues would disrupt the ACT structural integrity and block CoA binding as indicated by the dramatic reduction in yeast survival and the abolishment of Smc3 acetylation *in vivo* (Fig. 2G and H). The hEsco1 was crystallized as a dimer formed by interactions between the β hairpins^47^. However, this mode of dimerization was not observed in either our peptide-free or substrate-bound crystal forms. To define the oligomeric state of xEco2, we performed multi-angle light scattering (MALS) and determined the solution masses of the free and substrate-bound ACT domain to be 25.5 and 26.7 (+/−0.54) kDa respectively, indicating their monomeric states in our system (Supplementary Fig. S4A).

### Structural Basis of Substrate Motif Recognition

**Figure 3:**
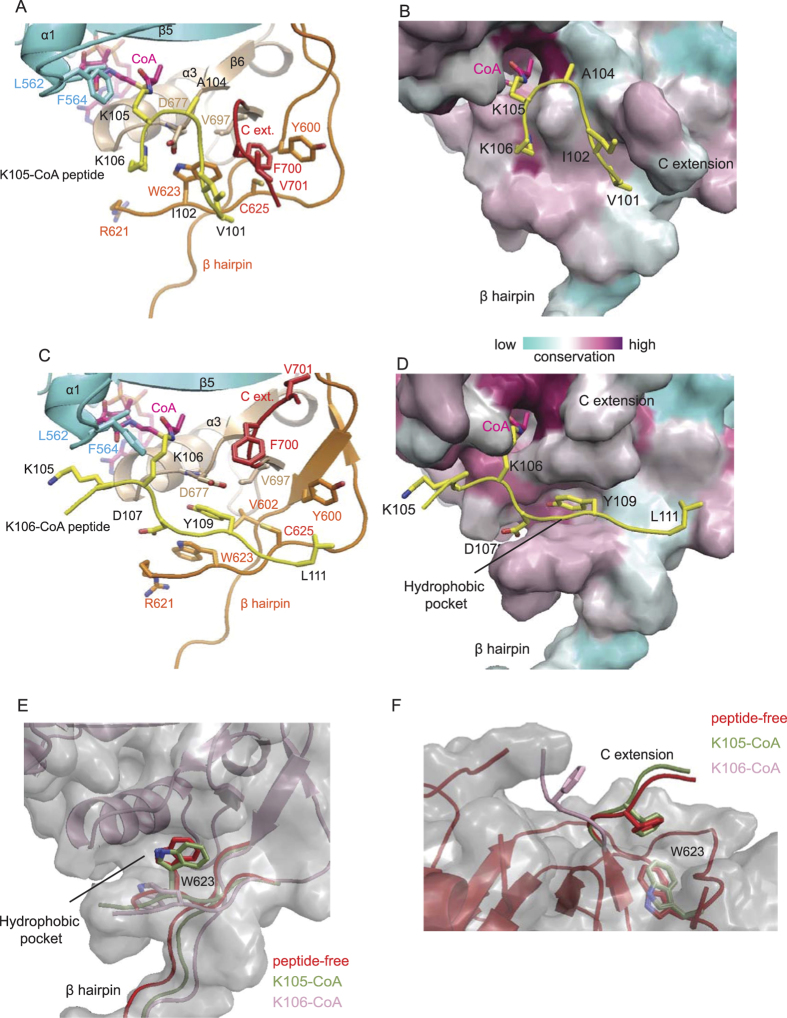
Substrate binding and conformational changes of the central β hairpin and C extension. (**A**) Details of xEco2-K105-CoA interaction showing substrate hairpin (yellow) coordinated by the C extension (red) in form of a β sheet. (**B**) Conserved surface rendition of K105-CoA bound to xEco2. (**C**) Details of xEco2-K106-CoA interaction showing substrate peptide (yellow) coordinated by the central β hairpin (orange) in form of a β sheet. (**D**) Conserved surface rendition of K106-CoA bound to xEco2. (**E**) Superimposition of peptide-free (red), K105-CoA (green), and K106-CoA (salmon) bound xEco2 structures showing occupation of and displacement of W623 from the central hydrophobic pocket. (**F**) Superimposition of the C extension in peptide-free (red), K105-CoA (green), and K106-CoA (salmon) bound conformations. The C extension shows a 180° flip from coordinating W623 at the hydrophobic pocket to an open conformation upon K106-CoA binding.

The xEco2-K105-CoA structure represents the faster acetylation event on *S. cerevisiae* K112 of Smc3. The peptide K105-CoA adopts a β turn conformation, which is readily accommodated by the substrate-binding site created by the extended loop connecting α1 to β3, the short loop connecting β6 to α3, the central β hairpin, and the C extension on the xEco2 structure (Figs 2D,E and 3A, and Supplementary Fig. 5). The side chain of K105-CoA, which protrudes from the CoA-binding cavity, is gated by the conserved L562 and F564 residues (Fig. 3A and B). The unmodified K106 salt-bridges to D677. This mode of coordinating K106 explains why a basic residue is favoured at P + 1 in the non-directional motif consensus (Fig. 1H). In addition, the conserved C extension of xEco2 forms a contiguous anti-parallel β sheet with residues 101–104 of the substrate hairpin, wherein I102 forms a hydrophobic contact with V701 (Fig. 3A).

Next we investigated the slower but biologically more important K106 acetylation of Smc3^8^, which is represented by our xEco2-K106-CoA structure (Figs 2F and 3C, and Supplementary Fig. 5). The substrate peptide adopts an extended β conformation different from K105-CoA binding but is accommodated by a conserved surface groove created by the same structural elements on xEco2 including the C-terminal loop of α1, the loop connecting β6 to α3, and the central β hairpin (Fig. 3C and D). Instead of the C extension, the binding of the K106 substrate peptide to xEco2 is mediated by main chain-side chain interactions through the formation of a parallel β sheet with the second strand of the central β hairpin.

The side chain of the K106-CoA conjugate protrudes from a central CoA-binding cavity gated by the conserved L562 and F564 residues (Fig. 3C and D), whose hydrophobic side chains also contact the methylene groups of K105 at position -1 (P − 1) and the methyl group of A104 at P − 2. At P + 1 from the acetylated lysine, the acidic side chain of D107 forms a salt bridge with the indole side chain of W623 on the central β hairpin. The structure rationalizes the propensity of Eco1 substrates to have an aliphatic residue at P − 1 and P − 2, and an acidic/polar residue at P + 1 flanking the target lysine in our substrate motif consensus (Fig. 1F). It is possible that the initial acetylation and amine neutralization of the P − 1 K105 increases its propensity to interact with the flanking L562 and F564 residues in a way that better mimics the leucine identified in our “forward” consensus motif. Unfortunately, we were unable to obtain diffracting crystals with the acetyl-K105-K106-CoA peptide, and therefore the possibility that acetyl-K105 binds in a conformation differing from our structure cannot be excluded.

### Identification of Catalytic Base

A recent study of the hEsco1 structure suggested that D107 of Smc3 acted as a general base for the nucleophilic attack on the target lysine during acetylation, similar to E570 in human PCAF^47^. However, in our xEco2-peptide K106 structure the Smc3 D107 interacts with the conserved R621 and W623 of xEco2 on the opposite side of the ε-amino group of the target lysine (Fig. 3C). It is possible that the nucleophilic attack is mediated by an alternatively conserved E594 in xEco2 (human E725), which positions its acidic side chain to the target lysine in the same manner as E570 of PCAF^48^,^49^ (Supplementary Figs 2A and 3B). Mutation of this residue has recently been reported to substantially reduce the acetylation activity of hEsco1^50^. The same study proposed that D810 might also act as a general base, though we note that this residue is not conserved in yeast Eco1, and further studies will be required to resolve this issue.

### K106 Peptide Binding Requires Changes at Central β Hairpin and C Extension

The binding of the K106-CoA peptide is accompanied by an outward tilt of the central β hairpin from its peptide-free conformation with W623 displaced from a central hydrophobic pocket by Y109 of the K106 peptide (Fig. 3C and E). This hydrophobic pocket is formed by a cluster of conserved hydrophobic residues V602 (β hairpin), W623 (β hairpin), C625 (β hairpin), and V697 (β7). Together with the aforementioned hydrophobic (P − 1) and acidic/polar (P + 1) binding sites, the anchoring of the substrate Y109 by the hydrophobic pocket positions the K106 sidechain optimally for acetylation (Fig. 3C). While equivalent individual β-hairpin mutations, W192A or M193A (*X. laevis* W623 or C625), of the hydrophobic pocket in yeast had little impact of Smc3 acetylation, combining both mutations or deleting the entire β hairpin (residues 189–198) drastically reduced yeast survival and eliminated Smc3 tandem lysine acetylation, confirming the importance of the β hairpin in Eco1 substrate recognition (Fig. 2G and H).

In the peptide-free xEco2 structure, the conserved W623 occupying the hydrophobic pocket is partially stacked against the conserved F700 on the C extension (Fig. 3F). The binding of peptide K106-CoA forces a 180° flip of the C extension, exposing the entire length of the substrate-binding groove (Fig. 3C and F). This mode of dramatic structural rearrangement between substrate-free and substrate-bound proteins is analogous to the T loop of Cdks switching from crisscrossing to exposing substrate-binding site during kinase activation^51^. The requirement for substantial conformational changes of K106 substrate binding compared to the readily accommodated K105 substrate by xEco2 may also explain why K105 acetylation is a faster and more rapid event (Fig. 1C and E). The interactions captured in our structures cover a very highly conserved region of Smc3 suggesting they can be applied to both the yeast and *Xenopus* systems used in our studies.

**Figure 4:**
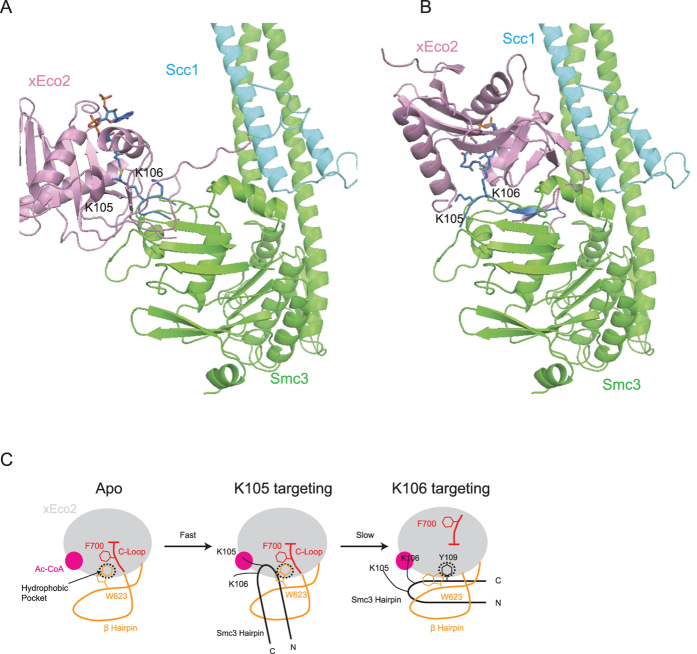
Mechanism of Eco1-mediated Smc3 acetylation. (**A**) Speculative model for Eco1-Smc3 interaction created by docking of xEco2-K105-CoA structure (salmon and blue) onto the *S. cerevisiae* Smc3-Scc1 (Smc3 is green; Scc1 is cyan) structure with reference to the relative positions of the tandem lysines (*S. cerevisiae* K112 and K113). (**B**) Speculative model for Eco1-Smc3 interaction created by docking of xEco2-K106-CoA structure (salmon and blue) onto the *S. cerevisiae* Smc3-Scc1 (Smc3 is green; Scc1 is cyan) structure with reference to the relative positions of the tandem lysines (*S. cerevisiae* K112 and K113). xEco2 rotates by 180° along the plane of the β sheet of Smc3 ATPase domain. (**C**) Schematics of Eco1 substrate specificity conferred by the concerted conformational changes of the central β hairpin (orange) and the C extension (red). The conserved hydrophobic pocket is occupied by W623 of xEco2 in the peptide-free and K105 targeting configurations, and by Y109 from Smc3 during K106 targeting respectively.

The conformations of the substrate peptides in both xEco2-K105-CoA and xEco2-K106-CoA structures are consistent with the β elements of the Smc3 ATPase domain crystal structure (PDB: 4UX3)^40^. Approximate positioning of both xEco2-peptide structures onto the yeast structure of Smc3-Scc1 by matching positions of the lysines and peptide backbone reveals that xEco2 is capable of binding both sites in Smc3 without any obvious clashes or changes in substrate peptide conformation (Fig. 4A and B). The transition between K105 and K106 acetylation is associated with a substantial increase of contact between xEco2 and Smc3. In fact, surface calculation using the PISA server^52^ shows that xEco2 and the K105 peptide (excluding CoA) has a protein-protein interface of 260 Å^2^ versus 486 Å^2^ between xEco2 and the K106 peptide. The smaller protein-protein interface could translate to a faster substrate release and turnover of *S. cerevisiae* K112 acetylation as seen in MS (Fig. 1C and E) and the weaker peptide density in xEco2-K105-CoA structure (compare Supplementary Fig. 4A and B).

## Conclusions

In this study, we examine the dynamics and substrate specificity of Eco1-mediated acetylation through the reconstitution of *in-vitro* acetylation assays coupled with mass spectrometry. We discover Eco1 substrate motif consensuses and the acetylation of Smc3 K112 to be a faster event prior to the slower but biologically important K113 acetylation. Furthermore, our xEco2-substrate structures illustrate that substrate specificity is conferred by the orchestrated conformational changes of the conserved central β hairpin and the C extension (Fig. 4C), rationalizing differential rates between the tandem lysine acetylation. These results, together with future studies examining the molecular consequences of substrate acetylation will provide new insights into the mechanism of multiple cellular pathways.

## Material and Methods

### Expression and Purification of Cohesin Subunits

*S. cerevisiae* cohesin (Smc1, Smc3, Rad21, and Scc3), Scc2-Scc4 complex, Pds5, and Wapl were amplified by polymerase chain reaction (PCR) using genomic DNA as templates and cloned into a modified pFBDM vector with a double Strep-tag II (ds) and a tobacco etch virus (TEV) cleavage site at the N terminus of Rad21, the C terminus of Scc2, the C terminus of Pds5, and the C terminus of Wapl respectively. The resultant protein expression cassettes were recombined with the DH10MultiBac cells to create the corresponding bacmids. *S. cerevisiae* cohesin was expressed using the baculovirus/insect cell (Sf21) systems whereas the Scc2-Scc4, Pds5, and Wapl were expressed using Hi5 insect cells. All proteins were purified by Strep-Tactin (Qiagen), anion exchange chromatography Porous Q, and S200 size-exclusion chromatography (GE Healthcare).

### *In-vitro* Acetylation of Cohesin Subunits

To obtain a consensus motif, Eco1 at a concentration of 70 μM was incubated with 0.7 μM of the following cohesin *S. cerevisiae* subunits: Scc2-Scc4, Pds5, and Wapl for 90 minutes at 37 °C. The reaction was carried out in 50 mM Tris pH 8, 50 mM NaCl, 0.5 mM DTT, 5 mM MgCl_2,_ and 1.5 mM Acetyl-CoA (Buffer A). 70 μM Eco1 was also incubated with 0.7 μM of Smc1-Sm3-Scc1-Scc3 in Buffer A with an addition of 1 mM ATP. The reactions were stopped and samples were analyzed by SDS-PAGE. The resulting protein bands were visualized by Coomassie blue staining and cut for MS analysis. To obtain more information on the tandem acetylation of Smc3’s K112 and K113, a time course experiment was conducted with Smc1-Smc3-Scc1-Scc3 in Buffer A with an addition of 1 mM ATP. Samples were taken at 0, 10, 20, and 40 minutes.

### Reductive Di-Methylation and PRM Assays

Gel pieces were cut into small gel plugs (1 mm^3^) and destained in 10 mM triethylammonium bicarbonate (TEAB), 40% acetonitrile at 37 °C. The solution was removed and the gel plugs were dehydrated with acetonitrile. 4 μl of 4% (v/v) formaldehyde (aq) and 4 μl 0.6 M sodium cyanoborohydride (aq) was added to 100 μl 10 mM TEAB, and transferred to the gel pieces for 1 h at 22 °C. The solution was removed, and the gel pieces were twice washed with 50 μl 1% ammonium hydroxide to quench any further reaction, after each wash the gel pieces were dried with acetonitrile. 50 μl 5% formic acid was then added to the gel pieces, removed and dried with acetonitrile. The gel pieces were washed four times in 10 mM TEAB and dried in acetonitrile.

500 ng of sequencing grade trypsin was added to each gel plug, rehydrated on ice and left to incubate overnight at 37 °C. Enough 10 mM TEAB was added to ensure that the gel pieces would not dry out. The solution was removed and transferred to a fresh tube, gel plugs were washed using 0.1% trifluoroacetic acid, 40% acetonitrile for 20 min and the solution added to the previous tube, finally the gel plugs were dehydrated with acetonitrile and again added to the tube. All extracted solutions were dried by vacuum centrifugation, before being resuspended in 50 μl 1% trifluoroacetic acid.

A Thermo Scientific Q Exactive mass spectrometer coupled to an UltiMate 3000 HPLC system for on-line liquid chromatographic separation was used for data acquisition. Each sample was analyzed in triplicate (10 μl per injection) and each LC-MS/MS run consisted of a 1 h gradient elution (75 μm × 50 cm C_18_ column). The samples were analyzed using PRM (Parallel Reaction Monitoring), with one full MS scan (*m*/*z* 400-1200, 70 000 resolution) being completed followed by six separate MS/MS events (HCD 27 NCE, 35 000 resolution, 200 000 AGC target) at *m*/*z* 574.31, 578.97, 583.63, 860.97, 867.95, and 874.94; this sequence was repeated throughout the total analysis. PRM chromatograms were then extracted using Skyline 3.5.0.9319 software.

### Generation of Eco1 Substrate Motif Consensuses

For Eco1 substrate motif generation, SDS-PAGE gel lanes containing cohesin complex and regulatory subunits treated or untreated with Eco1 were subjected to in-gel trypsin digestion. Peptide mixtures were analyzed on a LTQ-Orbitrap-Velos system using a top 10 data dependent acquisition method. Peptide identification was performed using MaxQuant v1.3 using default parameters and acetylation of lysines selected as a variable modification. The false discovery rate at the site level was controlled at 1% and the resulting acetyl(K).txt output file was used for further analysis using Perseus software. Acetylated peptides of cohesin subunits were categorized manually as forward (ϕ-AcK-B/Z) (n = 36), reverse (B/Z-AcK-ϕ) (n = 42), and no direction (n = 27). Eco1 substrate motif consensuses were subsequently generated using the iceLogo server^39^.

### Expression and Purification of xEco2 ACT Domain

xEco2 ACT domain was amplified (residue 523–702) and cloned into a pET-28a vector, which contained the addition of an N-terminal His_6_ tag and a TEV protease site. This vector was transformed using standard procedures into BL21(DE3)-RIL competent cells (Agilent). 1L of LB was inoculated with an overnight culture of cells grown at 37 °C. The 1L culture was grown until an OD of 0.6 was reached at which point, the cells were cooled to 16 °C. The expression xEco2 was induced by the addition of 0.25 mM IPTG for 18 hours. The harvested cells were sonicated in 50 mM Tris 8.5, 300 mM NaCl, 0.5 mM TCEP, 10% Glycerol and 10 mM imidazole and then centrifuged at 35000 × g, 4 °C for 1 hour. The following supernatant was loaded onto a 5 mL His-Trap HP (GE Healthcare) and the protein was eluted in a gradient of imidazole from 10 mM to 0.5 M. The protein containing fractions were loaded onto a pre-equilibrated MonoQ 10/300 GL (GE Healthcare) column in 20 mM Tris 8.5, 50 mM NaCl and 0.5 mM TCEP. xEco2 was eluted from the ion exchange in a 50 mM to 1M NaCl gradient. The protein peak was loaded onto a HiLoad 16/60 Superdex 75 column (GE Healthcare) equilibrated in 10 mM Tris 7.5, 150 mM NaCl and 0.5 mM TCEP. SDS-PAGE analysis was used to determine protein purity.

### Synthesis of Peptide-CoA Conjugate

Solid phase synthesis of peptides was carried out on an Intavis peptide synthesizer, starting with 50 μmole Rink Amide AM low loading resin, using N-Fmoc protected amino acids and HCTU as the coupling reagent (Merck Chemicals). At the site of lysine modification Fmoc-Lys(ivDde)-OH was incorporated and the N terminus of the peptides was acetylated. Following chain assembly, the ivDde group was removed by three treatments of the peptidyl-resin with 15 ml of 2% hydrazine/dimethylformamide (DMF) for 1 h at RT and subsequent washing with DMF and dichloromethane (DCM). Next the peptidyl-resin was reacted with 8.3 equivalents (eq) of R-2-bromopropionic acid and 8.3 eq of diisopropylcarbodiimide in DMF for 2 h at room temperature, followed by washing with DMF and DCM. Cleavage and deprotection of the peptide was achieved by adding the peptidyl-resin to 10 ml of 92.5% trifluoroacetic acid (TFA), 2.5% ethanedithiol, 2.5% triisopropyl silane, and 2.5% H_2_O. After 4 h, the resin was removed by filtration and peptides were precipitated with diethyl ether on ice. Peptides were isolated by centrifugation, dissolved in H_2_O and freeze dried overnight. Peptides were purified on a C8 reverse phase HPLC column (Agilent PrepHT Zorbax 300SB-C8, 21.2 × 250 mm, 7 m). Buffer A was 1% acetonitrile, 0.08% trifluoroacetic acid in H_2_O, buffer B was 90% acetonitrile, 0.08% trifluoroacetic acid in H_2_O. The elution gradient was from 10% to 50% buffer B, over 40 min at a flow rate of 8 ml/min. Conjugation of the purified bromopropionyl-peptide to Coenzyme A was achieved by adding 25 mg (5 eq) of Coenzyme A hydrate (Sigma) dissolved in 1M triethylammonium bicarbonate, pH 8.4–8.6 (Fluka) with 20 mg of peptide in 1 ml dimethylsuphoxide and stirring at room temperature for 18 h. Following lyophilization of the solution, the conjugated peptide was solubilized in H_2_0 and purified by RP-HPLC as previously described using a gradient of 0% to 40% Buffer B over 40 min. The peak fractions were analyzed by LC–MS on an Agilent 1100 LC-MSD. The calculated molecular weights of the peptide were in agreement with the mass found.

### Crystallization of xEco2 and Structure Solution

xEco2 ACT was concentrated to 15 mg/ml and crystallized alone or with the addition of the conjugated peptides K106-CoA or K105-CoA. The peptide-free form of xEco2 crystallized in 0.2 M NaCl, 0.1 M imidazole pH 7 and 0.864 M ammonium phosphate. Crystals were harvested with the addition of 30% ethylene glycol. K106-CoA and xEco2 crystallized in 0.18 M MgCl_2_, 10% glycerol, 27% 2-propanol and 0.09 M HEPES pH 7.5 and were harvested in 25% ethylene glycol. Peptide K105-CoA with xEco2 crystallized in 40% ethylene glycol, 0.1 M HEPES pH 7.5, 5% PEG 3K. Data for all crystal forms was collected at Diamond light source on beamline I02 or I03. For crystal of K106-CoA molecular replacement was carried out in Phaser^53^ using hEsco1 as a start model^47^. Phenix.autobuild^54^ was used to build an initial model and multiple rounds of rebuilding and refinement were carried out in Coot^55^ and phenix.refine^54^. The peptide-free and K105-CoA structures were solved using the K106-CoA structure as a start model in Phaser and multiple rounds of re-building and refinement were carried out as described. For both the peptide structures the peptide was built in the final stages of refinement. Statistics for the data collections and refinement are shown for all three structures in Table S2.

### *In-vivo* Assays

For the spot assays pMET-eco1AID (Y4766) cells were transformed with an empty URA3 plasmid or a URA3 plasmid carrying different versions of Eco1 expressed under Eco1 endogenous promoter. Serial dilutions of stationary cultures were dropped onto YNB-MET-URA as well as YPD + IAA plates and grown for 2 days at 30 °C. To determine the *in vivo* acetylation of Smc3 by mutant Eco1, cells were grown in YNB CSM-MET and arrested in G1 by addition of alpha factor 1 H. Prior to release, cells were transferred to YPD + IAA + alpha factor. Upon G1 arrest, cells were released into nocodazole for 90 minutes, when proteins were TCA extracted. 15 μg of proteins were separated on 10% SDS-PAGE, transferred and submitted to Western blotting with anti-Acetyl Smc3 (K. Shirahige) and anti HA (F7, Santa Cruz Biotechnology).

## Additional Information

**Accession codes:** Atomic coordinates and structure Fsu factors of xEco2, xEco2-K105-CoA, and xEco2-K106-CoA have been deposited in the Protein Data Bank under accession codes 5N1U, 5N1W, and 5N22 respectively.

**How to cite this article**: Chao, W. C. H. *et al*. Structural Basis of Eco1-Mediated Cohesin Acetylation. *Sci. Rep.* **7**, 44313; doi: 10.1038/srep44313 (2017).

**Publisher's note:** Springer Nature remains neutral with regard to jurisdictional claims in published maps and institutional affiliations.

## Supplementary Material

Supplementary Information

Supplementary Table S1
