# Erratum: Structural Basis of Eco1-Mediated Cohesin Acetylation

**DOI:** 10.1038/srep46382

**Published:** 2017-04-11

**Authors:** William C. H. Chao, Benjamin O. Wade, Céline Bouchoux, Andrew W. Jones, Andrew G. Purkiss, Stefania Federico, Nicola O’Reilly, Ambrosius P. Snijders, Frank Uhlmann, Martin R. Singleton

Scientific Reports 7: Article number: 4431310.1038/srep44313; published online: 03 14 2017; updated: 04 11 2017

This Article contains an error in the Additional Information section.

“Accession codes: Atomic coordinates and structure Fsu factors of xEco2, xEco2-K105-CoA, and xEco2-K106-CoA have been deposited in the Protein Data Bank under accession codes 5N1U, 5N1W, and 5N22 respectively”.

should read:

“Accession codes: Atomic coordinates and structure factors of xEco2, xEco2-K105-CoA, and xEco2-K106-CoA have been deposited in the Protein Data Bank under accession codes 5N1U, 5N1W, and 5N22 respectively”.

