# Supplementary material for: Structural Basis of Eco1-Mediated Cohesin Acetylation
Source: Sci Rep. 2017 Mar 14;7:44313. doi: 10.1038/srep44313 (PMC5349539; doi:10.1038/srep44313)
Supplement: Supplementary Information [file srep44313-s1.pdf]

SUPPLEMENTARY INFORMATION

Structural Basis of Eco1-Mediated Cohesin Acetylation

William C. H. Chao, Benjamin O. Wade, Céline Bouchoux, Andrew W. Jones, Andrew G. Purkiss, Stefania Federico, Nicola O'Reilly, Ambrosius P. Snijders, Frank Uhlmann, and Martin R. Singleton

Supplementary Figures S1 - S5
Supplementary Tables S1 - S2

Supplementary Figure S1. The PRM data reveals signal for acK112-meK113 but not for meK112-acK113.

A) Example PRM chromatograms of the transitions for the target peptide with the sequence TVGLKKDDYQLNDR containing a single acetylation and a single di-methylation. Co-elution of the transitions (fragments) and their relative peak areas confirm the identity of the peptide.
B) The same as (A) but now only displaying the chromatograms for the transitions indicative for acK112-meK113 (b5 and y9 ions).
C) The same as (A) but now only displaying the chromatograms for the transitions indicative for meK112-acK113 (b5 and y9 ions).

Supplementary Figure S2. Sequence alignment of Eco1/2 ACTs and location of temperature sensitive and Robert's Syndrome mutations.

A) Hydrophobic residues in blue interact with the methylene moiety of the target lysine side chain. Conserved glutamate in pink potentially catalyzes the nucleophilic attack on the ε-amino group of the target lysine. Basic residues in green interact with conserved D107 at P-1 of Smc3 peptide. Hydrophobic residues in yellow form the hydrophobic pocket, which accommodates Y109 in the K106 peptide. *S. cerevisiae ts* mutation G211D (red) in Eco1. Human Roberts Syndrome mutation W539G (orange) in hEsco1. F700 (wheat) in the C extension which determines the availability of the K106 substrate-binding groove.
B) Details of the equivalent *S. cerevisiae ts* mutation G211D (red) (xEco2 G635) and Roberts Syndrome mutation W539G (red) in hEsco1 (xEco2 W640).

Supplementary Figure S3. Electron density maps of the primary and secondary substrate peptides bound to xEco2.

A) Unbiased omit (Fo-Fc) map of the electron density for the K105 (left) and K106 (right) peptides contoured at 2σ and 3σ respectively. Peptide (but not Co-A) coordinates were excluded from the map calculation.
B) Feature enhanced maps[1] of the electron density for the K105 (left) and K106 (right) peptides contoured at 1σ. Peptide coordinates were excluded during map calculation.

Supplementary Figure S4. xEco2 oligomerisation and putative catalytic base.

A) MALS analysis showing solution masses for peptide-free and peptide bound xEco2 of 25.5 and 26.7 (+/- 0.54) kDa respectively. The peptide alone has a mass of 2.5 kDa.

B) Superimposition of xEco2 (salmon) with human PCAF (green) showing xEco2 E594 as equivalent to PCAF E570 to catalyze nucleophilic attack on the ε-amino group of the target lysine. K106-CoA peptide is in yellow.

Supplementary Figure S5. Details of substrate binding sites.

A) Expanded diagram of the K105 (left) and K106 (right) peptide binding sites with key interacting residues highlighted.

B) Ligplot[2] diagram summarising ligand-protein interactions for the K105 (left) and K106 (right) peptides. For clarity, the coenzyme has been excluded from the diagram.

SUPPLEMENTARY REFERENCES

1. Afonine, P. V. *et al.* FEM: feature-enhanced map. *Acta Crystallogr D Biol Crystallogr* **71,** 646–666 (2015).
2. Wallace, A. C., Laskowski, R. A. & Thornton, J. M. LIGPLOT: a program to generate schematic diagrams of protein-ligand interactions. *Protein Eng* **8,** 127–134 (1995).

Supplementary Table S1. Peptides identified by mass spectrometry.

Please see separate Excel file Supplementary Table S1.

Supplementary Table S2. Crystallographic statistics for all structures.

Chao and Wade et al. Sup. Figure 1

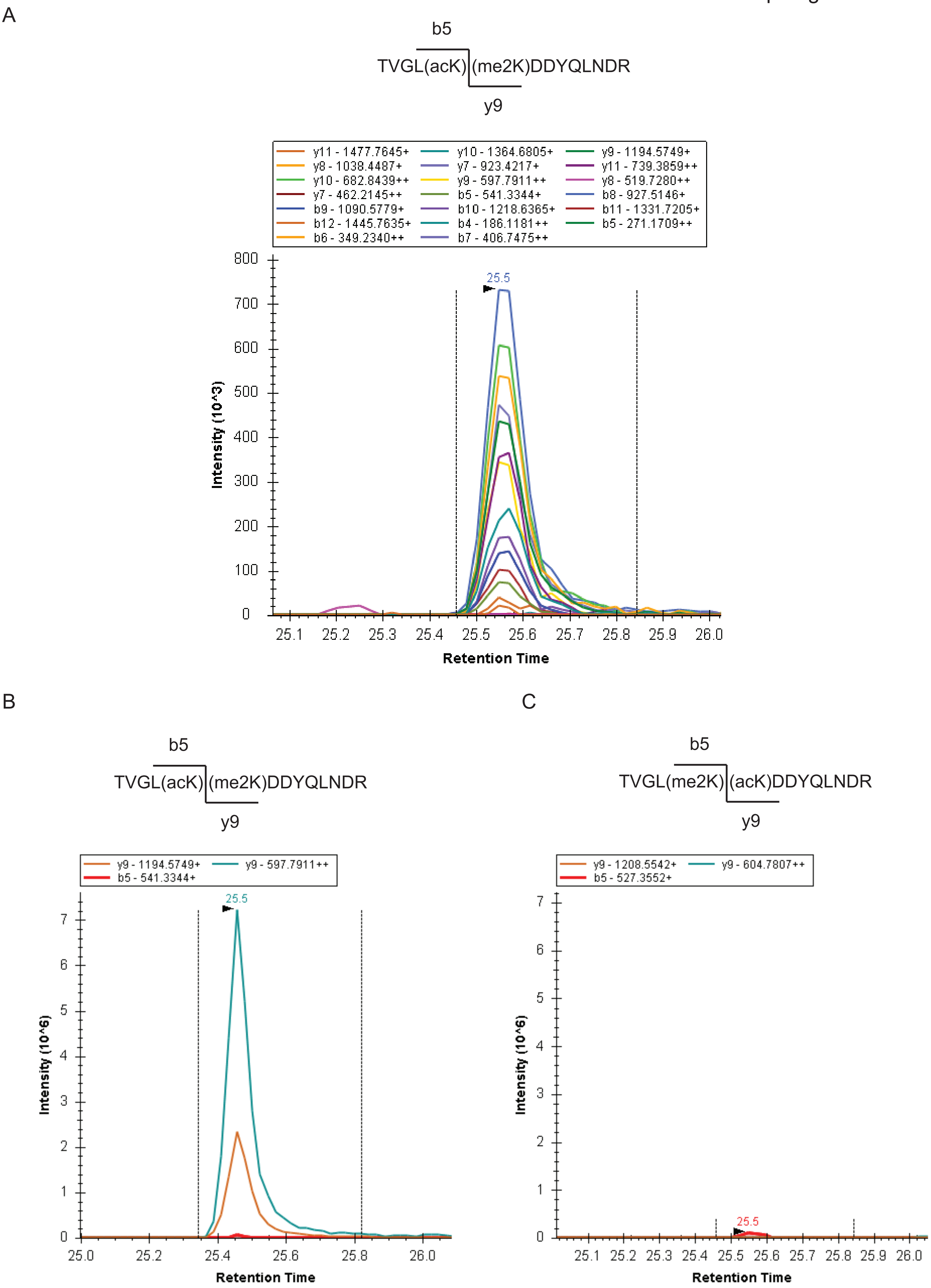

Chao and Wade et al. Sup. Figure 2

A

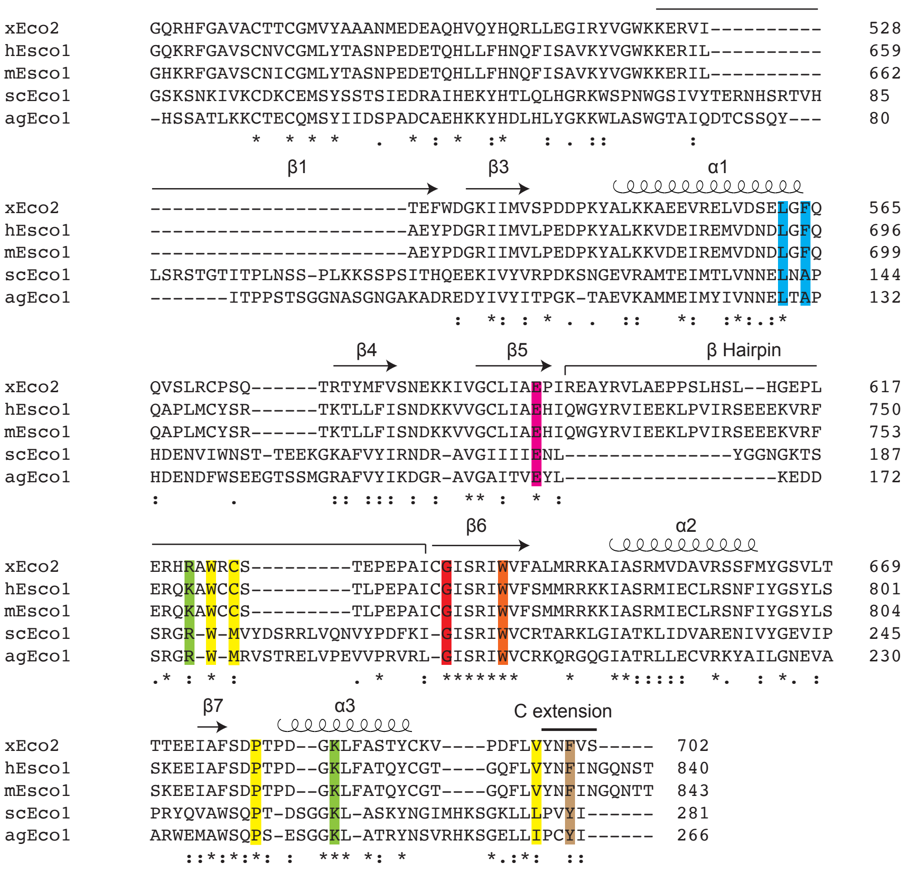

B

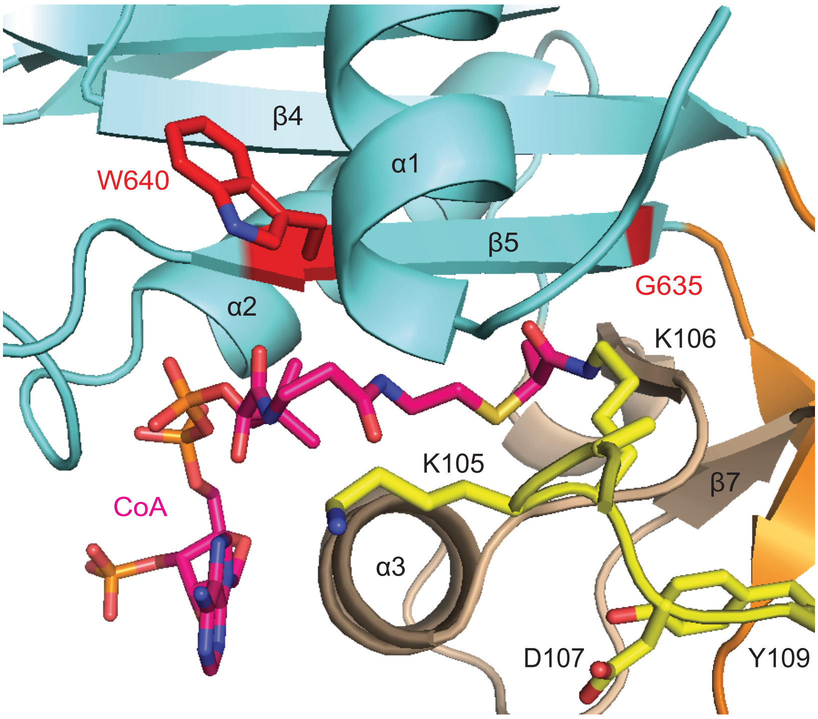

K106-CoA peptide

A

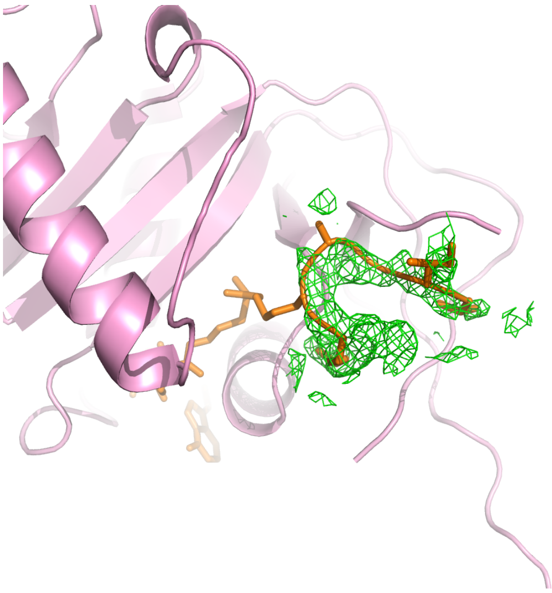

K105 Fo-Fc map, 2σ

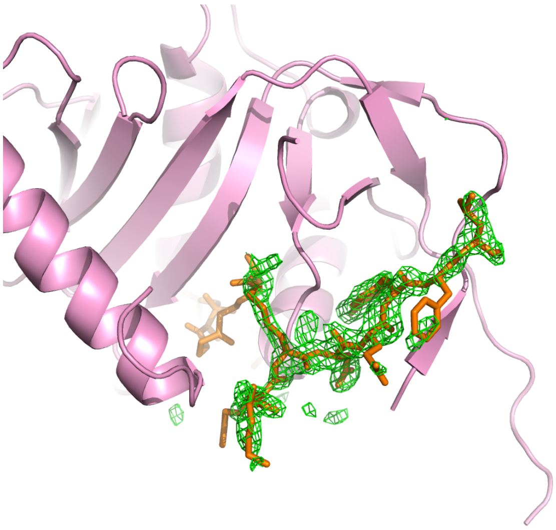

K106 Fo-Fc map, 3σ

B

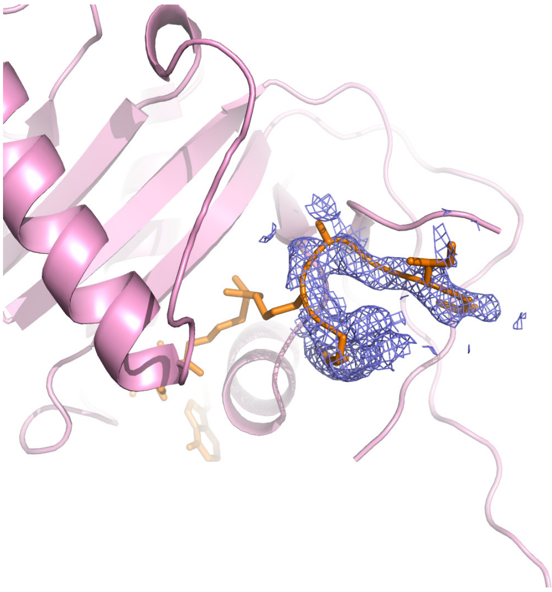

K105 FEM map, 1σ

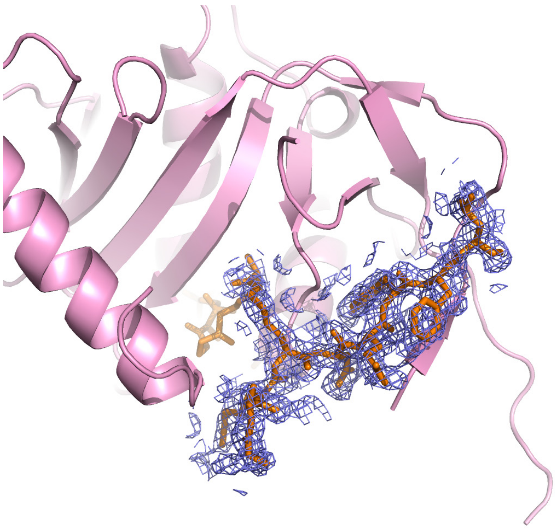

K106 FEM map, 1σ

A

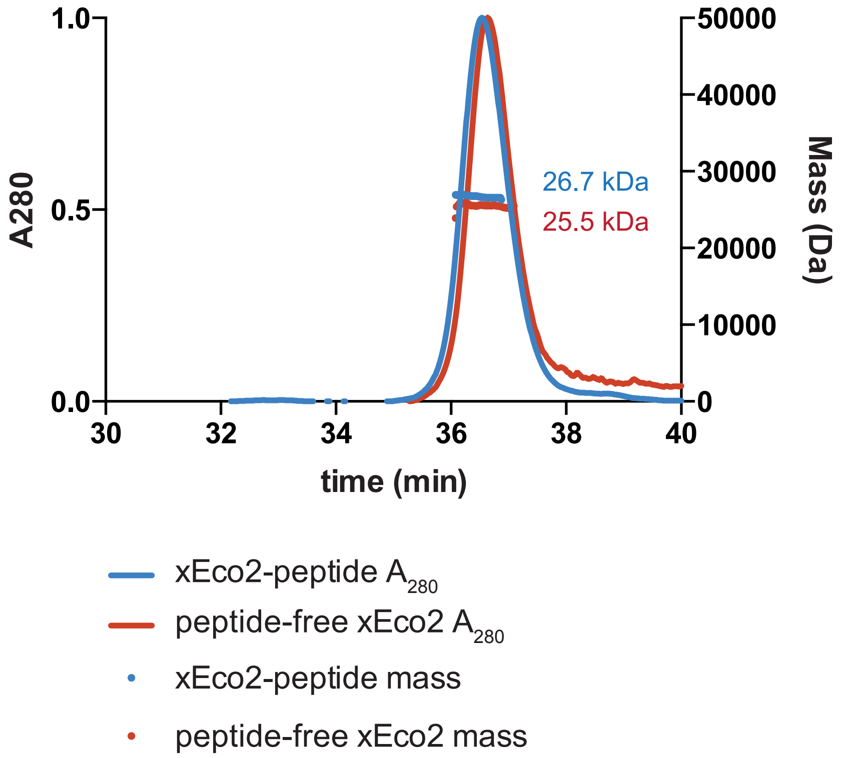
1.0
0.5
0.0
A280
50000
40000
30000
20000
10000
0
Mass (Da)
30
32
34
36
38
40
time (min)
26.7 kDa
25.5 kDa
xEco2-peptide A280
peptide-free xEco2 A280
xEco2-peptide mass
peptide-free xEco2 mass

B

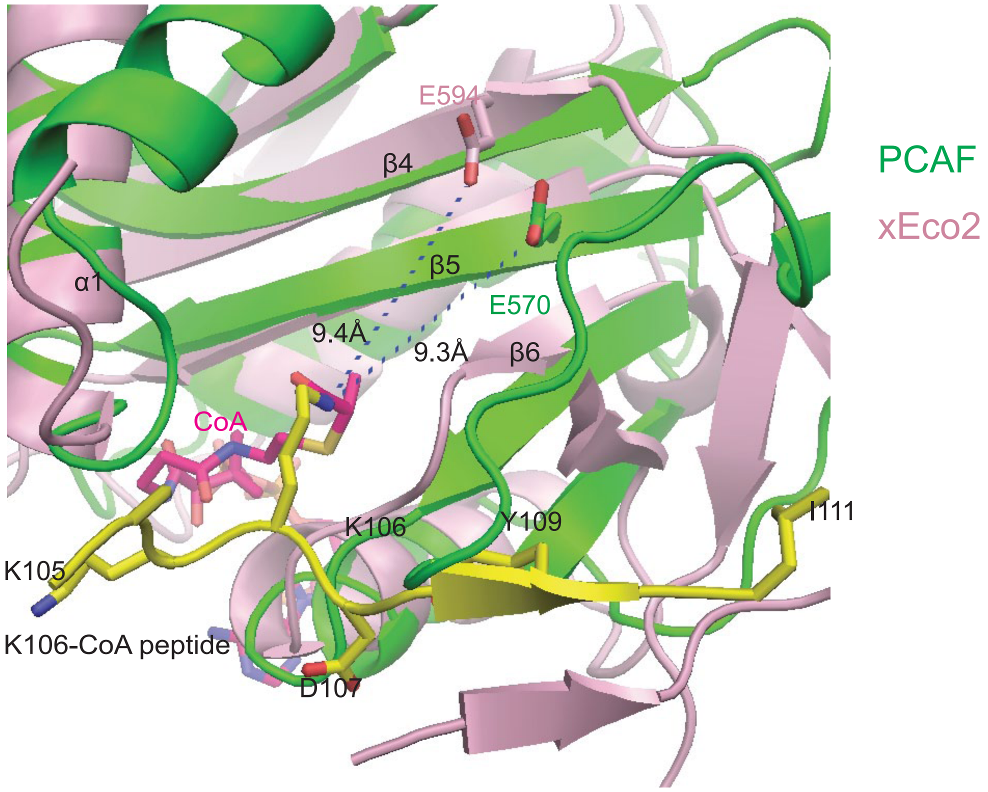
E594
β4
β5
α1
E570
9.4Å
9.3Å
β6
CoA
K106
Y109
I111
K105
K106-CoA peptide
D107
PCAF
xEco2

A

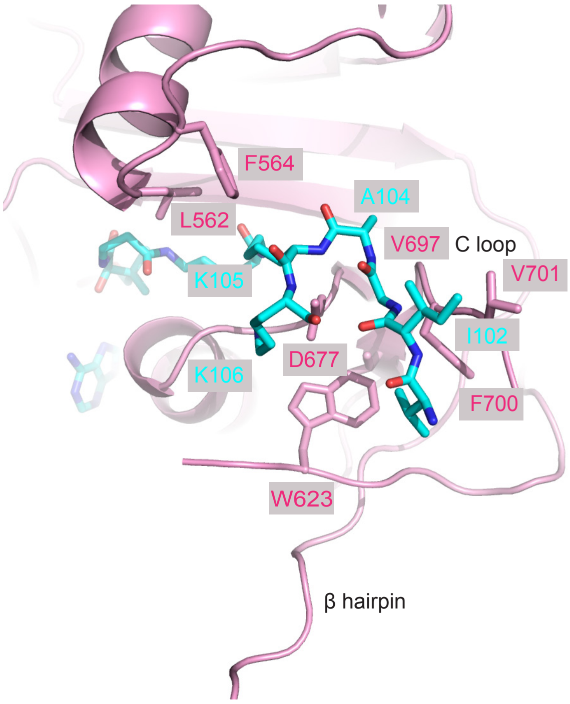

K105-CoA peptide

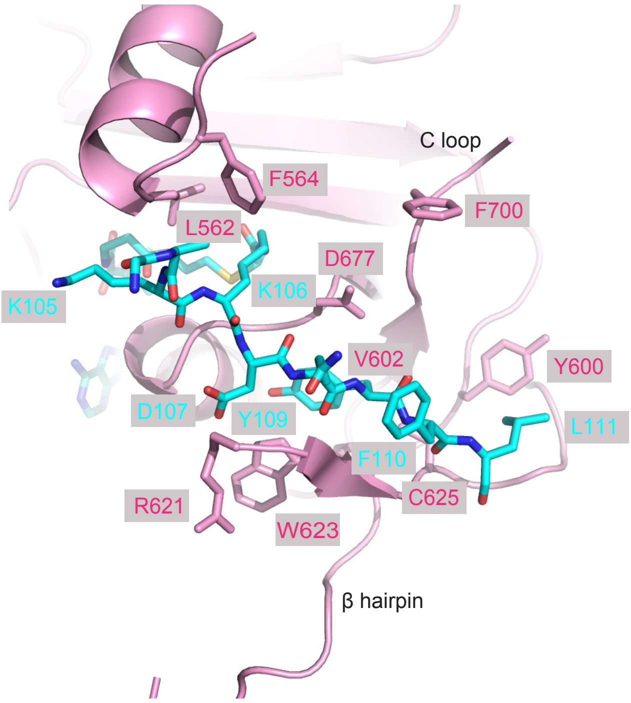

K106-CoA peptide

B

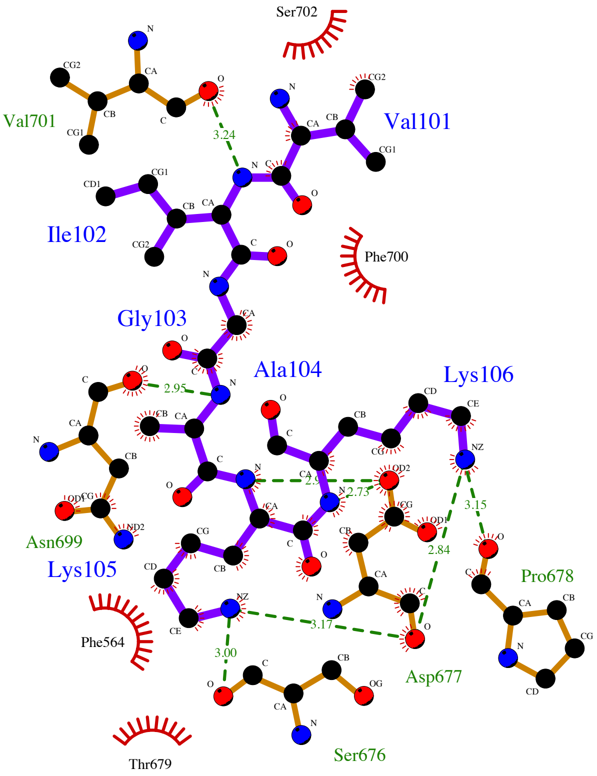

K105-CoA peptide

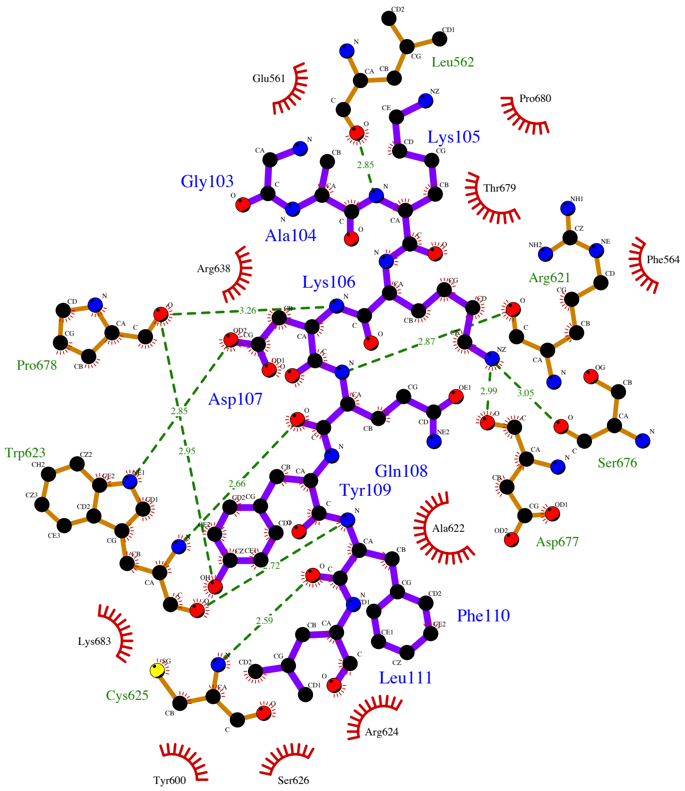

K106-CoA peptide

	K106-CoA	K105-CoA	Peptide-free
Wavelength	0.9795	0.9795	0.9795
Resolution range	59.16-1.99 (2.06-1.99)	46.97-2.3 (2.38-2.3)	28.83-2.98 (3.08-2.98)
Space group	$P2_1$	$P2_1$	$P2_12_12_1$
Unit cell (Å,°)	107.24 57.77 70.95 90 89.97 90	57.30 60.61 66.90 90 99.63 90	58.48 66.29 109.76 90 90 90
Total reflections	197266 (19580)	131452 (13235)	55624 (4904)
Unique reflections	59859 (5936)	20265 (2010)	9148 (860)
Multiplicity	3.3 (3.3)	6.5 (6.6)	6.1 (5.7)
Completeness (%)	0.98 (1.00)	1.00 (1.00)	0.99 (0.96)
Mean I/sigma(I)	8.25 (1.65)	6.78 (2.02)	7.70 (1.52)
Wilson B-factor	31.92	31.9	83.52
R-merge	0.06494 (0.7646)	0.2701 (1.834)	0.2968 (1.159)
R-meas	0.07758 (0.9127)	0.2943 (1.992)	0.3252 (1.27)
CC1/2	0.998 (0.615)	0.98 (0.639)	0.968 (0.449)
CC*	0.999 (0.873)	0.995 (0.883)	0.992 (0.787)
Reflections used in refinement	58984 (5707)	20227 (2001)	9089 (837)
Reflections used for R_{free}	2817 (270)	988 (99)	908 (83)
R_{work}	0.208 (0.3658)	0.2102 (0.3180)	0.2247 (0.3684)
R_{free}	0.2374 (0.3866)	0.2297 (0.3884)	0.2669 (0.3897)
CC(work)	0.955 (0.811)	0.948 (0.841)	0.956 (0.547)
CC(free)	0.939 (0.721)	0.909 (0.684)	0.899 (0.566)
Number of non-hydrogen atoms	6005	2999	2725
macromolecules	5497	2719	2629
ligands	244	124	96
Protein residues	689	348	340
RMS(bonds)	0.007	0.005	0.007
RMS(angles)	0.77	0.63	0.88
Ramachandran favored (%)	97	95	94
Ramachandran allowed (%)	2.7	4.5	6.1
Ramachandran outliers (%)	0.6	0.3	0.3
Rotamer outliers (%)	3.8	2.4	5.7
Clashscore	3.67	3.71	7.06
Average B-factor	45.96	44.17	72.81
Macromolecules	46.41	44.67	73.25
ligands	35.36	33.44	60.61
solvent	46.39	44.09	
Number of TLS groups	23		

SUPPLEMENTARY TABLE S2. Crystallographic Statistics
